# The outcome of out-of-hospital cardiac arrest based on the etiology of cardiac arrest; A scoping review

**DOI:** 10.1371/journal.pone.0330083

**Published:** 2025-08-11

**Authors:** Sedigheh Shaeri, Julie Considine, Katie N. Dainty, Theresa Mariero Olasveengen, Laurie J. Morrison

**Affiliations:** 1 Institute of Health Policy, Management, and Evaluation, Dalla Lana School of Public Health, University of Toronto, Toronto, Canada; 2 Centre for Research and Quality, SickKids Hospital, Toronto, Canada; 3 School of Nursing and Midwifery and Centre for Quality and Patient Safety Research in the Institute for Health Transformation, Deakin University, Geelong, Australia; 4 Centre for Quality and Patient Safety Research - Eastern Health, Box Hill, Australia; 5 Office of Research & Innovation, North York General hospital, Toronto, Canada; 6 Department of Anesthesia and Intensive Care, Oslo University Hospital, and Institute of Clinical Medicine, University of Oslo, Oslo, Norway; 7 Department of Emergency Services, Sunnybrook Health Sciences Centre, Toronto, Canada; 8 The Division of Emergency Medicine, Department of Medicine, University of Toronto, Toronto, Canada; Azienda Ospedaliero Universitaria Careggi, ITALY

## Abstract

**Background:**

Disparity is believed to exist between generic Utstein etiological classifications of 2004 and 2015 when compared with confirmed etiologies, but the impact of this disparity on reported survival outcomes is unknown.

**Objective:**

This scoping review was proposed with two objectives: 1-to report outcomes based on confirmed etiology of OHCA in comparison with Utstein classified etiologies and 2- to identify outcomes of OHCA by etiology following cause-targeted interventions.

**Method:**

Medline, Embase, and EBM- Cochrane databases were searched from inception to 2024. Studies were selected if included population was adults with OHCA for whom survival outcomes of OHCA were reported based on the confirmed etiology compared to Utstein etiological classification (2004 or 2015) or reported based on the etiology following cause-targeted interventions. A descriptive review of included studies was conducted.

**Result:**

The search yielded 24,833 citations. Thirty-nine studies met inclusion criteria. These articles were predominantly published in Europe and North America between 2010–2024. The Utstein etiological classification was used in all studies (Utstein 2004; n = 31, Utstein 2015; n = 8). Survival to discharge was higher for drug overdose induced OHCA than presumed cardiac etiologies (9–83% vs 8.3–63%). For confirmed etiology of drowning, 30-day survival was higher than the rate reported by presumed cardiac etiologies (Utstein 2004) (11.5% vs 8.8%) while survival to discharge was lower following confirmed etiologies of trauma (1.7–5.1% vs 8–12%), hanging (3.3–43% vs 12–61%), respiratory disease (5% vs 9%), and intracranial hemorrhage (ICH) (11% vs 40%) compared with Utestin etiological classifications (2004 or 2015). Thrombolysis therapy resulted in better 30-day survival compared to placebo for OHCA due to presumed pulmonary embolism (16% vs 6%; p = 0.05), and conventional resuscitation resulted in higher survival for OHCA due to drowning (10.5% vs 8.6%) or respiratory disease (6.8% vs 4.54%) versus chest compression only.

**Conclusion:**

The reported survival outcomes for confirmed non-cardiac etiologies is inconsistent across studies compared with Utstein etiological classifications of 2004 or 2015. Better survival outcomes following few cause-targeted interventions may be attributed to etiologically well-defined patient cohorts. More vigorous case selection based on etiology may refine the reported outcomes and comparisons with interventions across published studies.

## Introduction

Globally, out of hospital cardiac arrest (OHCA) occurs around 51–107 cases per 100,000 people annually [1,2], but the one-year survival following OHCA remains low at about 8% (95% CI 5.8–9.5%) [3]. Out-of-hospital cardiac arrest occurs for various underlying reasons, and identifying the etiology of cardiac arrest is sometimes challenging for prehospital personnel who treat the patient initially, and for up to 50% of patients with an unsuccessful resuscitation who are pronounced dead and left at scene [2,4–6]. The Utstein etiological classification for OHCA was first defined in 2004 and revised in 2015. The two broad generic classifications defined in 2004 of “presumed cardiac” and “obvious non-cardiac” were replaced with even broader terms “medical” and “non-medical” categories in 2015 [7]. The “medical” group was heterogeneous and consisted of confirmed cardiac, non-cardiac, and all unknown etiologies [7].

A prior scoping review identified a three- fold difference between the proportion of initial generic Utstein presumed cardiac classification of OHCA and confirmed cardiac etiologies [8]. This misclassification may affect reporting survival outcomes of observational studies. Two previous studies demonstrated that the survival outcome analysed before and after an intervention was significantly changed after adjusting the denominator from cases with presumed cardiac etiology (Utstein 2004) to include only confirmed cardiac etiologies. The denominator was reduced from 85% to 56%, and the estimated primary outcomes were changed from 24.6% to 18.3% before an intervention and from 31% to 33.8% after an intervention [9,10]. This misclassification may also affect randomized trial results, if the population with a confirmed diagnosis that is more likely to benefit from the intervention is mixed in with a generic population defined by the broader Utstein classification of presumed cardiac (2004) or medical (2015).

More than 80 clinical trials have been conducted in prehospital settings, but only 23 (28%) demonstrated significant results [11]. While some studies that recruited patients with “presumed cardiac”, “no obvious”, and “non-traumatic” inclusion criteria demonstrated neutral outcomes [12–15], studies with etiologically well-defined inclusion criteria directly linked to the intervention (e.g., refractory ventricular fibrillation (VF) and defibrillation or use of an antiarrhythmic drug) were more likely to show a difference [16,17]. Further, many studies have been conducted to evaluate the survival outcome of OHCA due to cardiac and non-cardiac etiologies. The previous systematic review further suggested presumed cardiac etiologies (Utstein 2004) had better ROSC (33.9% vs 21.5%), survival to admission (23.5% vs 19.3%), survival to discharge (10% vs 7%), one-month survival (10.5% vs 6.7%), and one-year survival (7.9% vs 7.1%) outcomes when compared with OHCA reported by generic etiology classification of non-traumatic etiologies [3], but to our knowledge, the survival outcome following each confirmed etiology of OHCA against presumed cardiac or medical etiologies has not been systematically investigated.

The aim of this scoping review is to explore all relevant published research to identify the current state of reported OHCA outcome based on each confirmed etiology compared with “presumed cardiac” (Utstein 2004) or “medical” (Utstein 2015) etiologies. Outcomes of interest will include standard outcomes such as return of spontaneous circulation (ROSC), admission to hospital, discharge from hospital, and favorable neurological outcomes. This scoping review will also investigate the reported survival outcome of OHCA by the etiology following prehospital cause-targeted interventions. The scoping review was considered the most appropriate method because the evidence was anticipated to be diverse and indirect [18].

## Method

Arksey and O’Malley’s methodological steps for scoping reviews, with the refinements proposed by Levac guided the protocol and conduct of this scoping review [18,19]. The international database of prospectively registered systematic reviews in health and social care (PROSPERO), Medline, Google Scholar, and open science framework were checked to confirm that no systematic, scoping, or narrative reviews on a similar topic have been published or were registered as an incomplete systematic review with PROSPERO.

This scoping review was conducted in accordance with the Preferred Reporting Items for Systematic Reviews and Meta-Analyses extension for Scoping Reviews (PRISMA-ScR) (S1 Appendix) [20]. The reviewer followed the written protocol and applied data screening steps and a standard data abstraction form available on https://www.covidence.org [21]. The protocol is registered with Open Science Framework (OSF) at https://doi.org/10.17605/OSF.IO/HTYN7 [22]. Our scoping review consisted of the following steps:

### Identifying the research question

The research questions were: What was the outcome following OHCA due to each confirmed medical and non-medical etiologies in the available published research in comparison with Utstein etiological classifications of presumed cardiac (Utstein 2004) or medical (Utstein 2015) etiologies? What were the outcome differences of prehospital interventional trials or studies reported by etiology?

### Search strategy and sources of evidence

The search strategy was developed by an experienced information specialist (DL) and applied to Medline, Excerpta Medica database (Embase), and Evidence-Based Medicine (EBM) review- Cochrane from inception to June 28, 2024. (Key search terms and search strategy: S2 Appendix)

### Eligibility Criteria

Table 1 explains the inclusion criteria for selecting articles. The population was limited to adult patients (as defined in each paper) who had experienced OHCA and were treated by emergency medical services (EMS) providers and for whom the initial diagnosis was assigned. The intervention was any prehospital etiologically specific or cause-targeted intervention that was applied to a cohort where the underlying etiologies of OHCA were likely confirmed (i.e., thrombolysis for pulmonary embolism or chest compression with or without ventilation for etiologies involving anoxia) (not necessary). The primary outcome of interest was to identify any clinical outcome (Return of Spontaneous Circulation (ROSC), survival to admission, survival to discharge, and favorable neurological outcomes) based on underlying etiology of OHCA. The second outcome of interest was to identify any outcome reported for patients with OHCA due to medical or non-medical etiologies following any prehospital cause-targeted interventions.

**Table 1:** Inclusion criteria for selecting studies.

Population: All adult patients (as defined in each paper) who had experienced out-of-hospital cardiac arrest (OHCA) and were treated by emergency medical services (EMS) personnel and for whom the initial diagnosis was assigned.
**Intervention:** Any prehospital cause-targeted intervention that was applied to a predefined etiology of OHCA. (not necessary)
**Comparator:** Outcome of OHCA reported by generic classifications of presumed cardiac (Utstein 2004) or medical (Utstein 2015) etiologies.
**Outcomes:** The **primary outcome** of interest was to identify any clinical outcome (Return of Spontaneous Circulation (ROSC), survival to admission, survival to discharge, and favorable neurological outcomes) based on assigned etiology of OHCA. The **second outcom**e of interest was to report comparative studies or trials where the interventions were unique to an etiology and outcomes reported based on the etiology.
**Study designs:** Any population-based comparative studies, randomized controlled trials (RCTs), experimental, and observational studies. Guidelines, editorial reviews, conference abstracts, and commentaries were excluded.
**Timeline and Language:** All published literature in **English** from **database inception** to **2024**.

### Study selection

All citations were uploaded into the Covidence website for screening. All duplicates were excluded. First, the primary author (SS) reviewed all titles and abstracts against inclusion and exclusion criteria. For initial screening, limited exclusion criteria were employed in order to have broader inclusion. After initial screening, all potential eligible full texts were retrieved and further reviewed by the primary author (SS) against the same eligibility criteria. Additional citations were found through hand search of the reference list of included studies following the initial review. Whenever there was uncertainty about a potential eligible study, the senior author (LJM) provided an additional review to verify selected full texts, and final decisions were achieved by discussion and consensus. The primary author reviewed the final selected articles multiple times to ensure the accurate selection.

### Data extraction and charting the data

The primary author (SS) followed the protocol and the JBI (Joanna Briggs Institute) methodological guideline [23] to extract the following data: 1- year of publication, 2- country of origin, 3- study design 4- population characteristics, 5- number of included population 6- etiology of OHCA, 7- source of initial and final etiologies if reported, 8- cause-targeted intervention if applied, 9- outcome of OHCA, 10- other related information.

### Collating and synthesizing data

To explain explicitly the result of this scoping review, all studies were grouped based on the etiology of OHCA. Descriptive analyses (e.g., numbers and percentages) were computed to present the prevalence of each characteristic of included studies. Country of origin and total number of included cohorts were mapped by using RStudio version 4.4.2 (ggplot2 package) to illustrate the geographical region of each included study [24,25]. A descriptive review was provided to explain the result of this review. No formal appraisal of the quality of the evidence was undertaken according to the methodology of scoping reviews [18,23].

## Result

### Study selection

**Fig 1:**
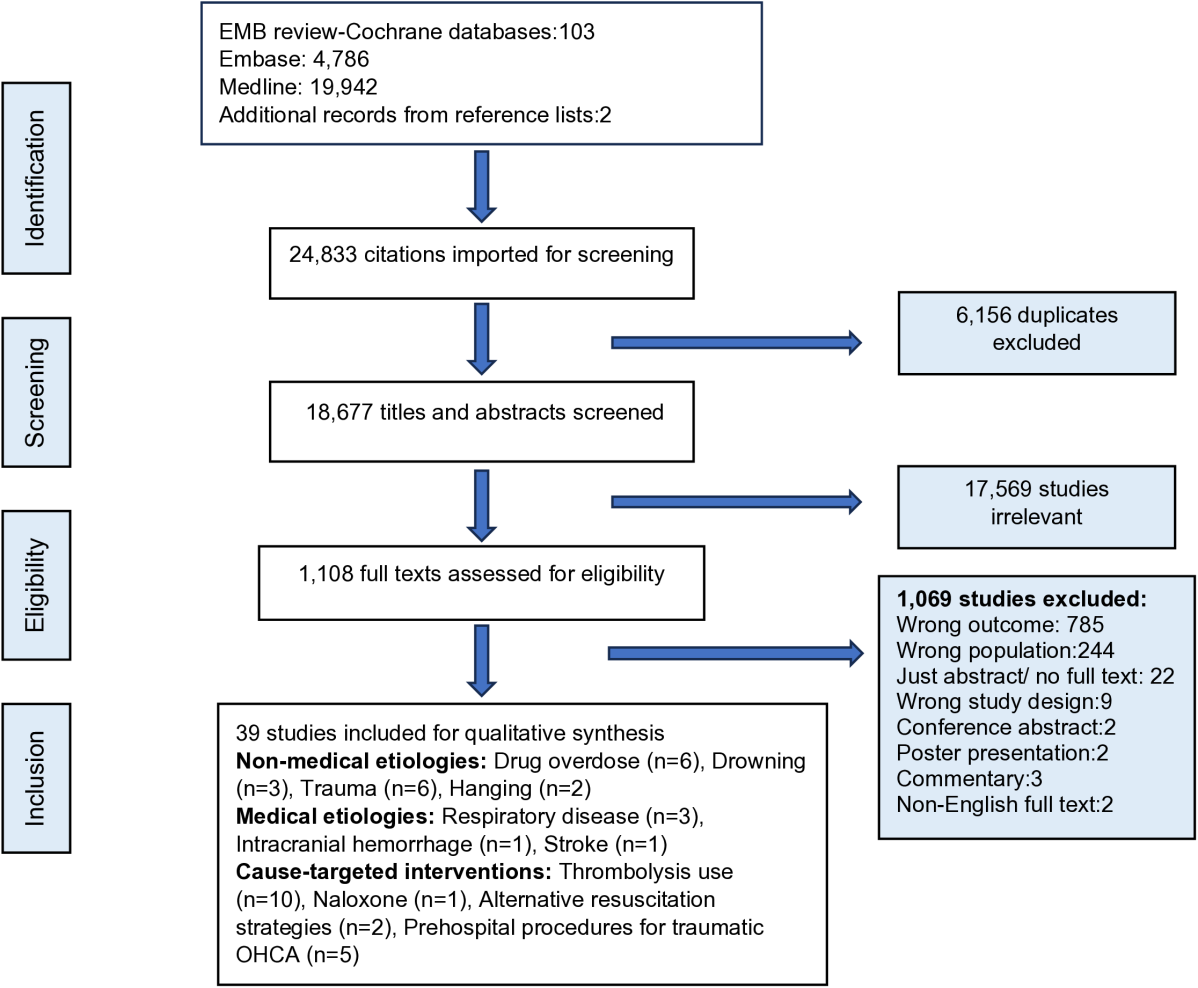
PRISMA flowchart. **EBM**: Evidence-based medicine. **Embase**: Excerpta Medica Database. **OHCA**: Out-of-hospital cardiac arrest.

The search yielded 103 citations from evidence-based medicine (EBM review-Cochrane) 4,786 citations from excerpta medica databases (Embase) 19,942 citations from Medline, and 2 from hand searching of bibliographies. After removal of duplicates (n = 6,156), 18,677 citations were screened for titles and abstracts. Fig 1 explains the process of selecting articles.

### Studies characteristics

**Fig 2:**
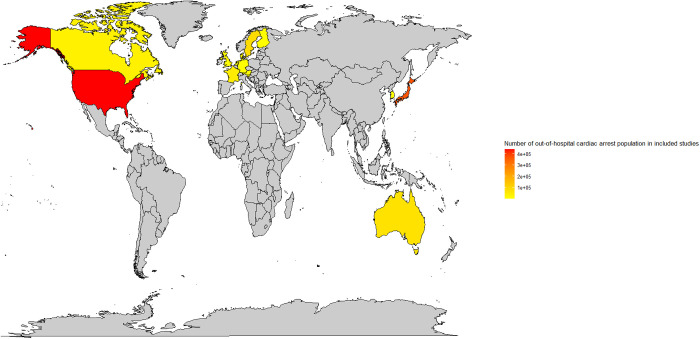
Map showing the geographical origin and included population of out-of-hospital cardiac arrest of studies that informed this review. The world map was created by RStudio using ggplot2 package. Fig 2: Map showing the geographical origin and included population of out-of-hospital cardiac arrest of studies that informed this review © 2025 by Sedigheh Shaeri. et al is licensed under CC BY 4.0.

**Fig 3:**
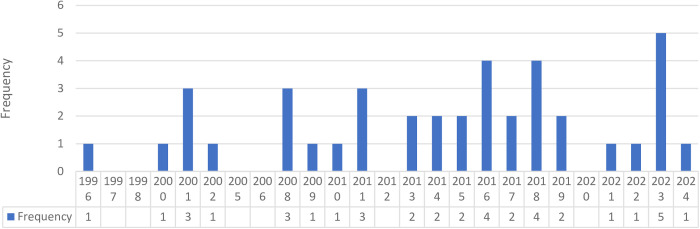
Year of publication of included studies.

In total, 39 studies met eligibility criteria and were included in this review. S3 and S4 Appendices present the summary of the study general characteristics and reported survival outcomes for each included study. The country of origin of included studies was mapped (Fig 2). The year of publication was shown graphically in Fig 3. These studies were grouped based on objectives of this review into the following groups: Group **1**–22 articles addressed the outcome of OHCA based on 1) the underlying non- medical etiologies, including drug overdose (n = 6) [26–31], drowning (n = 3) [32–34], trauma (n = 6) [35–40], and hanging (n = 2) [41,42], and 2) medical etiologies, including respiratory diseases (n = 3) [43–45], intracranial hemorrhage (ICH) (n = 1) [46], and stroke (n = 1) [47]. Group **2**–18 comparative studies or trials focused on prehospital cause-targeted interventions unique to an etiology, including the use of thrombolysis treatment for presumed cardiac etiologies (n = 7) [48–54] and pulmonary embolism (PE) (n = 3) [55–57], naloxone (n = 1) [58], different cardiopulmonary resuscitation strategies (n = 2) [44,59], and prehospital interventions for trauma induced cardiac arrest (n = 5) [60–64]. One study addressed both objectives and was included in both Group 1 and 2 [44]. The descriptive characteristics of included articles are presented in Table 2.

**Table 2:** Descriptive characteristics of included studies.

	Number	%
**Origin country:**		
**Europe**	20	51.2%
France [38,45,49,55,56]	5	
Sweden [32,37,43,60]	4	
Austria ^[^51,57]	2	
UK [36]	1	
Germany [52]	1	
Slovenia [33]	1	
Finland [48]	1	
Multicentre [50]	1	
Netherland [61]	1	
Denmark [40,62]	2	
**North America**	10	25%
USA [26–28,30,31,58,64]	7	
Canada [29,34,53]	3	
**Asia**	6	15.3%
Japan [44,47,59,63]	4	
South Korea [42,46]	2	
**Australia [**35,39,41**]**	3	7%
**Type of Study:**		
Observational [26^–^ 49^,^ 51,52,54–64]	37	95%
Randomized control trial [50,53]	2	5%
**Articles grouped based on the etiology of OHCA:**		
**Non-medical etiologies:**	**17**	
Drug overdose	6	
Drowning	3	
Hanging	2	
Trauma	6	
**Medical etiologies:**	**5**	
Respiratory disease	3	
ICH and Stroke	2	
**Cause-targeted interventions:**	**18**	
**Thrombolysis therapy:**	**10**	
Presumed cardiac etiologies	7	
Pulmonary embolism	3	
**Naloxone**	**1**	
**Alternative resuscitation strategies**	**2**	
**Prehospital procedures for traumatic OHCA**	**5**	

**ICH**: Intracranial hemorrhage. **OHCA**: Out-of-hospital cardiac arrest.

### Outcomes of OHCA by confirmed etiology

Table 3 summarizes included studies that reported survival outcomes of OHCA by confirmed etiology in comparison with Utstein etiological classifications (Utstein 2004 or 2015).

**Table 3:** Summary of observational studies reporting survival outcomes after OHCA by confirmed etiology and Utstein classifications.

Author	Etiology as confirmed vs Utstein classification	Number of patients	Reported survival outcome; %, p-value (95% Confidence Interval, OR, or RR, when reported. When not reported, cell is left blank)^^^				
			ROSC%	Hospital admission%	Hospital discharge%	30-day survival%	Functional Neurological Outcome CPC 1 or 2^&^, or MRS ≤ 2^$^;%
**Drug overdose (OD)**							
Koller [26]	OD	180	25%	34%	18.9% OR:3.89 (1.91–7.92) p < 0.001		
	Presumed cardiac^**^	2162	26.4% p = 0.6	31% p = 0.4	11.3% p = 0.014		
Elmer [27]	OD	85			83%		13% CPC 1 or 2 at discharge
	Presumed cardiac ^**^	506			63% p = 0.03		17% CPC 1 or 2 at discharge p = 0.51
Salcido [28]	OD	1351	33% OR:1.55 (1.35–1.77)	OR:1.76 (1.49–2.04)	12.7% OR:2.35 (1.72–2.64)		
	Presumed cardiac ^**^	54,921	30% p = 0.05		8.9% p = 0.05		
Orkins [29]	OD	344	20%	20.11%	9.52% OR:1.44 (1.15–1.81)		8.11% MRS ≤ 2
	Presumed cardiac ^**^	18,595	20.11% p = 0.4	19.8% p = 0.9	8.3% p = 0.41		8.49% MRS ≤ 2 p = 0.81
Yogeswaran [31]	OD	702			20% OR:1.59 (1.23–2.04)		18% CPC 1 or 2 at discharge OR:1.65 (1.26–2.14)
	Non-OD	6,088			18%^*^		17% CPC 1 or 2 at discharge^*^
Shekhar [30]	OD	29,500					15.2% CPC 1 or 2 at discharge
	Presumed cardiac ^**^	338,073					6.9% CPC 1 or 2 at discharge p < 0.001
**Drowning**							
Claesson [32]	Drowning	225		28.7%		11.5%	
	Presumed cardiac ^**^	7494		21.2% p = 0.006		8.8%^*^	
Grmec [33]	Drowning	32	65%	60%	44%		85% CPC 1 or 2 at discharge
	Presumed cardiac ^**^	528	57%*	48%*	22% p = 0.01		61% CPC 1 or 2 at discharge p = 0.08
Buick [34]	Drowning	98		27.8**%**	5.1%		
	Presumed cardiac ^**^	14,093		18.1% p = 0.013	5.9% p = 0.75		
**Trauma**							
Deasy [35]	Trauma	2,187	15.4%		5.1%		
	Presumed cardiac ^**^	24,284	30.9% p < 0.001		12% p < 0.001		
Beck [39]	Trauma	1,354		7.40%	1.7%		
	Medical^***^	16,076		16% p < 0.001	8.7% p < 0.001		
Barnard [36]	Trauma	304		19.3% (95% CI 15.3–24.2%)	3.8% (95% CI 2.1–6.8%)		
	Non-traumatic	8,805		27.9%* (95% CI 27–28.9%)	8% (95% CI 7.4–8.7%) p < 0.012		
Djarv [37]	Trauma	1,774				3.7%	
	Medical^***^	72,547				8.1% p < 0.01	
Escutnaire [38]	Trauma	3,209				1.5% OR: 0.24(0.18–0.32)	
	Medical^***^	40,878				5.9% p < 0.001	
Walthers [40]	Trauma	984	27.3%	24.2%		7.2%	
	Non-Traumatic	29,231	32.3%, p = 0.001	27.4% p = 0.032		14.2% p < 0.001	
**Hanging**							
Deasy [41]	Hanging	1321	29.3%	5.7%	3.3%		
	Presumed cardiac ^**^	24284	30.9% p < 0.001	12.5% p = 0.03	12% p < 0.001		
Shin [42]	Hanging	105			43%		6% CPC 1 or 2 at one month
	Presumed cardiac ^**^	859			61% p < 0.001		29% CPC 1 or 2 at one month p < 0.001
**Respiratory diseases**							
Herlitz [43]	Respiratory	130			5%		
	Presumed cardiac^**^	2867			9%^*^		
Fukuda [44]	Respiratory	7071	9.7%			6%	1.7% CPC 1 or 2 at one month
	Presumed cardiac^**^	67245	8.1%			6.3%	3.8% CPC 1 or 2 at one month
	Others	45,940	8.6% p < 0.0001			4.8% p < 0.0001	1.7% CPC 1 or 2 at one month p < 0.001
Orban [45]	Respiratory	95					19%CPC 1 or 2 at ICU discharge
	Presumed cardiac ^**^	156					42%CPC 1 or 2 at ICU discharge p < 0.001
**Intracranial hemorrhage and stroke**							
Shin [46]	ICH	92	96%	83%	11%		0 CPC 1 or 2 at one month
	Non-ICH	712	94% p = 0.5	80% p = 0.5	40% p < 0.001		16.4% CPC 1 or 2 at one month p < 0.001
Fukuda [47]	Stroke	18,682	9.9%			3.6%	1.2% CPC1 or 2 at one month
	Presumed cardiac^**^	224,458	5.9%			4.9%	2.6% CPC 1 or 2 at one month
	Confirmed cardiac	66,571	9.6% p < 0.0001			8.9% p < 0.0001	5.7% CPC 1 or 2 at one month p < 0.0001

**CI**: Confidence Interval. **CPC**: Cerebral Performance Category. **MRS**: Modified Rankin Score. **ICH**: Intracranial Hemorrhage. **OD**: Drug Overdose. **OR**: Odd Ratio. **ROSC**: Return of Spontaneous Circulation. **RR**: Relative Risk.

^ All studies did not report percentage, p-value, OR, RR, or 95% CI for all survival outcome intervals.

& CPC1: Good cerebral performance. CPC 2: Moderate cerebral disability.

$ MRS 1: No significant disability. MRS 2: Slight disability.

* p-value not reported.

** Presumed cardiac (Utstein 2004).

*** Medical (Utstein 2015).

### Drug overdose

In total, six studies reported the outcome of OHCA due to drug overdoses (OD) [26–31]. The etiology of drug overdose was based on EMS databases (e.g., history of drug use, witnessed arrest, or evidence of drug paraphernalia), some general characteristics suggesting drug overdose (e.g., young cohort and non-shockable rhythm), medical charts, and toxicology tests [26–31]. These articles defined drug-overdose OHCA if the patients consumed any drugs, including illicit or recreational drugs, opioid, and methadone prior to cardiac arrest [26–31]. One study excluded alcohol overdose cases [26].

In total, 32,162 patients with OHCA due to drug overdose (OD) were compared with 429,345 patients with presumed cardiac etiologies [26–31]. Five studies reported patients with OHCA due to drug overdose had better survival to hospital discharge than presumed cardiac OHCA [26–29,31] (Table 3).

### Drowning

Three studies examined the outcome of OHCA due to drowning (n = 385) compared to presumed cardiac etiologies (n = 22,115) [32–34]. All studies reported presumed etiologies (Utstein 2004) of OHCA were derived from EMS data using the Utstein style [32–34]. The etiology of drowning was confirmed by in-hospital documentation [33,34]. (S3 Appendix) Two studies reported confirmed drowning etiology had higher survival to hospital discharge (44% vs 22%; p < 0.01) [33], hospital admission (28.7% vs 21.2%;p = 0.006), and one month survival (11.5% vs 8.8%) [32] when compared with presumed cardiac classification (Utstein 2004). One study failed to demonstrate a significant difference in survival to hospital discharge for drowning versus presumed cardiac etiologies (5.1% vs 5.9%; p = 0.75) [34] (Table 3).

### Trauma

In total, five cohort studies focused on the outcomes of trauma-related OHCA [35–40]. The etiology of trauma was assigned based on EMS documentation in data registry according to Utstein 2004 or 2015 classifications and defined as cardiac arrest that occurs due to penetrating, blunt, and burn injuries [35–40] (S3 Appendix).

Three studies compared the survival of patients with traumatic OHCA (n = 6,337) with patients with OHCA of medical etiologies (Utstein 2015) (n = 129,501) [37–39]. Three studies compared the outcome of traumatic OHCA (n = 3,475) with presumed cardiac (Utstein 2004) and all non-traumatic etiologies (n = 62,320) [35,36,40]. Patients with medical, non-traumatic, or presumed cardiac OHCA had better survival to hospital discharge than those with traumatic etiology [35–40] (Table 3).

### Hanging

Two studies discussed the outcome of hanging- induced OHCA [41,42]. The etiology of hanging was based on Victorian ambulance cardiac arrest registry (VACAR) and Korean hypothermia network (KORHN) out-of-hospital data registry and confirmed with hospital records [41,42] (S3 Appendix).

These studies analysed survival to hospital discharge among patients with hanging- related OHCA (n = 1,426) compared with OHCA of presumed cardiac (Utstein 2004) etiologies (n = 25,143). Patients with OHCA due to hanging had lower survival to hospital discharge (3.3%vs 12%) [41] (43% vs 61%; p < 0.001) [42] and poorer neurological recovery (6% vs 29%; p < 0.001) [42] than patients with OHCA from presumed cardiac etiologies (Table 3).

### Respiratory diseases

Three studies compared the outcome of OHCA due to respiratory disease (n = 7,296) with OHCA from presumed cardiac (Utstein 2004) etiologies (n = 70,268) [43–45]. The initial etiology was derived from EMS data in the Utstein format, but the final etiology was retrieved from medical charts [43–45]. One study defined inclusion criteria and considered all the following diagnoses as a respiratory etiology of OHCA: asthma attack, pneumonia, aspiration, pulmonary embolism, chronic obstructive pulmonary disease (COPD), hanging, and drowning [45] (S3 Appendix).

OHCA patients with presumed cardiac etiologies had better survival to hospital discharge (9% vs 5%) [43], 30-day survival (6.3% vs 6%; p < 0.0001) [44] and favorable neurological outcomes compared with respiratory disease etiologies (3.8% vs 1.7%; p < 0.001) [44] (79% vs 21% p < 0.001) [45] (Table 3).

### Intracranial hemorrhage (ICH) and stroke

One study evaluated the outcome of OHCA after confirmed stroke (n = 18,682) with presumed cardiac (Utstein 2004) (n = 224,454), and another study examined the outcome of OHCA after confirmed intracranial hemorrhage (ICH) (n = 92) compared with outcome of non-ICH OHCA (n = 712) [46,47]. The etiology of OHCA was determined through reviewing in-hospital charts following brain CT scan [46,47]. No specific definition was provided for stroke cases. ICH cases were defined as all patients with subarachnoid hemorrhage (SAH), ICH, epidural hemorrhage, and subdural hemorrhage diagnoses based on brain CT scan criteria and not due to head trauma [46].

Patients with confirmed ICH had poor survival to hospital discharge with poor neurological recovery compared with non-ICH etiologies [46]. Lower survival outcome was observed with stroke etiology than presumed cardiac (Utstein 2004) and confirmed cardiac etiologies [47] (Table 3).

### Outcomes after cause-targeted interventions

In total, 18 studies investigated prehospital cause-targeted interventions. These studies were grouped based on the cause-targeted intervention, including use of thrombolysis, naloxone, different resuscitation strategies, and prehospital interventions to treat traumatic injuries. Some studies targeted the population most likely to benefit from intervention whereas other studies targeted a broad population as defined by Utstein classification. Reported survival outcomes by each included study are summarized in Table 4.

**Table 4:** Summary of reported survival outcomes for OHCA by targeted intervention and etiology.

Author	Design of Study	Etiology	Interventions^$^ N	Reported survival outcomes; N (%), p-value (95% Confidence intervals, OR, or RR, when reported; when not reported cell is left blank) ^^^				
				Return of spontaneous circulation(ROSC)	Hospital admission	Hospital discharge	30-day survival	Favorable neurological outcome; CPC1or 2^*^
**Thrombolysis therapy**								
Abu-Laban [53]	Randomized Controlled Trial	OHCA with PEA	TPA:112	25(21.4%)	7(6%)	1(0.9%)		
			Control:112	27(23.3%) p = 0.85	6(5.2%) p = 0.99	0 p = 0.99		
Bottiger [50]	Randomized Controlled Trial	Presumed cardiac (Utstein 2004)	Tenecteplase:525	283 (55%)	281(53%)	78(15.1%)		41(47%) CPC 1 or 2 at DC or 30 days
			Placebo:525	279(54%) RR:1.01 (0.9–1.13) p = 0.96	289 (55%) RR:0.97(0.8–1.09) p = 0.6	90 (17%) RR:0.86 (0.65–1.14) p = 0.33		45(46.9%) CPC 1 or 2 at DC or 30 days RR:1.02 (0.75–1.38)^**^
Voipio [48]	Observational	Acute Myocardial Infarction	Streptokinase:17	17(100%)				7(41%) CPC 1 or 2 at DC
			Alteplase:24	21(87%)				8 (33%) CPC 1 or 2 at DC
			Reteplase:18	16(88%)**				6 (33%) CPC 1 or 2 at DC^**^
Renard [49]	Observational	Non-traumatic	Fibrinolytic (Yes):107		51(47.7%) OR:2.9(1.9–4.4)			
			Fibrinolytic (No)= 1,154		272 (23.6%) p < 0.001			
Bottiger [54]	Observational	Unsuccessful resuscitation within 15 min	RTPA (Yes): 40	27(68%) OR:2.65(1.11–6.25)	23(58%) OR:3.15(1.32–7.69)	6(15%)		
			RTPA (No): 50	22(44%) p = 0.026	15(30%) p = 0.02	4(8%) ^**^		
Lederer [51]	Observational	Non-traumatic	RTPA (Yes):108	70 (70%)		27(25%)		
			RTPA(No):216	110(51%) p = 0.01		33(15.3%) p = 0.048		
Arntz [52]	Observational	Acute Myocardial Infarction (ST-elevated myocardial infarction	Thrombolysis after ROSC:50		35(70%)	23(46%)		
			Thrombolysis during CPR:3		2(66%)^**^	1(33%)^**^		
Kurkciyan [57]	Observational	Pulmonary Embolism	Thrombolysis (Yes):12	10(83%)		12(16%)		
			Thrombolysis (No):8	3(37%)^**^		0^**^		
Bougouin [55]	Observational	Pulmonary Embolism	Thrombolysis (Yes):40			30% OR:12.5 (1.8–89) p < 0.01		
			Thrombolysis (No):34			15%		
Javaudin [56]	Observational	Pulmonary Embolism	Thrombolytic (Yes):58				23(16%)	6(10%) CPC 1 or 2 at one month
			Thrombolytic (No):188				2(6%) p = 0.05	9(5%) CPC 1 or 2 at one month RR:1.97 (0.7–5.56) ^**^
**Naloxone**								
Saybolt [58]	Observational	Presumed opioid overdose	Respond to Naloxone^***^ (yes):15		4(27%) (95% CI 4–49)	1(7%) (95% CI −6–19)**		
			no response:21		not reported	0		
**Conventional CPR vs chest compression only vs no CPR**								
Fukuda [44]	Observational	Respiratory	Conventional CPR:757	98(12.90%)			52(6.87%)	16(2.11%) CPC 1 or 2 at one month
			Chest compression only:2,403	197(8.2%) OR:0.6 (0.47–0.78) p = 0.0002			109(4.54%) OR:0.64 (0.46–0.91) p = 0.013	32(1.3%) CPC 1 or 2 at one month OR:0.63 (0.35–1.17) p = 0.13
			No CPR:3,911	393(10.05%) OR:0.75 (0.6–0.95) p = 0.02			266(6.8%) OR:0.99 (0.73–1.36) p = 0.9	70(1.8%) CPC 1 or 2 at one month OR:0.84 (0.50–1.51) p = 0.55
Fukuda [59]	Observational	Drowning	Conventional CPR:968	98(10.6%)			97(10.5%)	70(7.5%) CPC 1 or 2 at one month
			Chest compression only:4,153	83(8.9%) RR:1.18 (0.89–1.15) p = 0.2			80(8.6%) RR:1.21 (0.9–1.16) p = 0.17	61(6.6%) CPC 1 or 2 at one month RR:1.15 (0.82–1.16) p = 0.4
**Prehospital procedures after traumatic cardiac arrest**								
Houwen [61]	Observational	Trauma	Thoracotomy; Yes:63 No:852			2(5.6%) 34(94.6%)^**^		
			Chest decompression Yes:514 No: 401			11(32.4%) 23(67%)^**^		
			Red packed cell transfusion: Yes: 94 No: 804			6(16.7%) 34(83%)^**^		
Ohlen [60]	Observational	Trauma	Thoracic decompression: Yes:66 No = 190				4(22.2%) 14(77.8%) p = 0.94	
Smida [64]	Observational	Trauma	Chest decompression Yes:820 No: 4,122	OR:0.79(0.55–1.14)				
Nagasawa [63]	Observational	Trauma	Resuscitative thoracotomy Yes:215 No:507	20 (9.3%) OR:0.32(0.15–0.7) 133 (26.2%)^**^				
Wolther [62]	Observational	Trauma	Chest decompression: Yes:110 No:112	27(24.5%) OR:0.35(0.13–0.91) 25(22.3%) ^**^			5(4.5%) 4(3.5%)^**^	

**CI**: Confidence Interval. **CPR**: Cardiopulmonary resuscitation. **CPC**: Cerebral performance category. **DC**: Discharge. **OHCA**: Out-of-hospital cardiac arrest. **OR**: Odd ratio. **PEA**: Pulseless electrical activity. **RTPA**: Recombinant tissue plasminogen activator. **RR**: Relative ratio. **TPA**: Tissue plasminogen activator.

$ Number of patients who received or not received the intervention.

^ All studies did not report number, percentage, p-value, OR, RR, or 95% CI for all survival outcome intervals.

* CPC1: Good cerebral performance. CPC 2: Moderate cerebral disability.

** p-value not reported.

*** Response to Naloxone: Patients showed reaction and ECG rhythm changes (e.g., asystole to PEA or PEA to VF) following naloxone injection.

### Thrombolysis therapy

#### Population defined as presumed cardiac (Utstein 2004) etiology.

Seven studies examined the association between thrombolytic treatment during CPR for 6,047 patients with OHCA of presumed cardiac (Utstein 2004) etiology (1,071 patients received thrombolysis vs 5,146 patients with no thrombolysis) [48–54]. Two studies refined inclusion criteria for presumed cardiac OHCA as presumed MI-related OHCA based on ST-elevation myocardial infarction (STEMI), or other factors suggesting MI such as left bundle branch block (LBBB) [48,52] whereas one trial randomized based on presumed cardiac etiology [50], and the second trial randomised based on presumed cardiac etiology with pulseless electrical activity [53].

In four studies, the initial documented OHCA etiology derived from EMS data was compared to confirmed OHCA etiology documented in autopsy reports [48,49,52,53]. Reported frequency of confirmed diagnoses of acute MI or cardiovascular disease (CVD) was varied across studies (94% [48], 59% [53],and 78% [52]) following autopsy or hospital admission in non-survivors and survivors, respectively. However, autopsy was performed infrequently in non-survivors across all studies with a reported range of 18–41% [49,53]. Two studies included patients with OHCA with presumed etiologies of AMI, PE, primary arrhythmia, and other non-cardiac, but did not report confirmed etiologies of OHCA [50,51] (S4 Appendix; Table 4).

One multicentre randomized controlled trial (RCT) reported lower survival to hospital discharge in patients with OHCA of presumed cardiac etiologies who received thrombolytic intervention compared with placebo group (15.1%vs 17.5%; p = 0.33) [50]. The second RCT demonstrated that there was no significant difference in survival to hospital discharge in patients with PEA as the initial rhythm who received tissue plasminogen activator (tPA) versus no tPA (0.9% vs 0; p = 0.99) [53] (Table 4).

#### Pulmonary embolism (PE).

Three studies evaluated the outcome of OHCA due to presumed PE following thrombolysis injection [55–57]. The etiology of OHCA was derived from EMS data (in the Utstein style) and France national OHCA registry and confirmed through autopsy and medical chart review of non-survivors and survivors, respectively [55–57]. The diagnosis of PE was presumed based on history of deep vain thrombosis (DVT), symptoms, and ECG changes prior to cardiac arrest or post ROSC. The diagnosis was verified by echocardiogram, spiral computed tomography (CT), ventilation-perfusion scan, or autopsy [55–57] (S4 Appendix).

In total, thrombolysis was administered to 110 out of 483 patients with presumed PE- induced OHCA during or after resuscitation in a prehospital setting [55–57]. Survival to hospital discharge was significantly higher among patients who received thrombolysis compared with patients who did not (16% vs 0) [57] (30% vs 15%) [55]. One study reported patients with thrombolysis had better odds to survive to hospital discharge following thrombolytic intervention (OR:12.5 (1.8–89; p < 0.01)) [55]. One observational study reported higher 30-day survival (16% vs 6%; p = 0.05) with no significant difference in favorable neurological outcome for patients receiving thrombolysis (10% vs 5%; RR:1.97; 95% CI:0.7–5.5) [56] (Table 4).

#### Naloxone.

One study involved patients who received naloxone for OD-related OHCA and compared the characteristics of patients who showed ECG rhythm changes (i.e., asystole to PEA or PEA to VF) following naloxone injection (defined as responders) with those patients who did not show any changes (defined as non-responders) [58]. This study reported better survival to hospital discharge among responders vs non-responders (7% vs 0; 95% CI −6–19%) [58] (Table 4).

#### Conventional CPR vs compression only vs no CPR.

The population of interest for these comparisons are etiologies where it is perceived that ventilation offered by conventional CPR may affect outcome. The etiology of OHCA was retrieved from EMS data registries and confirmed with emergency physicians collaborating with EMS and medical chart review [44,59] (S4 Appendix).

One observational study investigated the association between different bystander CPR strategies (conventional CPR vs compression only vs no CPR) in OHCA due to drowning with favorable neurological outcome at one-month [59]. This study found no statistically significant difference at discharge between chest compression-only and conventional CPR for cases confirmed for drowning etiology [59]. However, in the same study, one-month survival and neurological outcomes were slightly higher among patients with a drowning etiology who received conventional CPR compared with patients who received chest compression-only (10.5% vs 8.6%; p = 0.17 and 7.5% vs 6.6%; p = 0.4, respectively) [59] (Table 4).

Another study examined the effect of chest compression-only vs conventional CPR vs no CPR for OHCA due to respiratory disease etiology [44]. This study identified that conventional CPR was associated with higher ROSC and one-month survival compared with no CPR and chest compression only CPR [44] (Table 4).

#### Prehospital interventions for traumatic OHCA.

The etiologic classification of traumatic cardiac arrest is probably the least contentious. It is unlikely to see an intervention unique to trauma resuscitation evaluated in a generic Utstein etiologic classification. In total, five studies investigated the association of prehospital thoracotomy, thoracic decompression (thoracostomy), and blood transfusion with outcome of OHCA due to trauma [60–64]. Trauma was defined as cardiac arrest that occurs following blunt, penetrating, burning injury, or traffic accident according to the Utstein template [60–64]. One study detailed the underlying etiology of cardiac arrest among survivors (n = 36) following traumatic injury, including hypoxia following brain injury (45%), hypovolemia (24%), cardiac tamponade (12%), hypoxia due to other etiologies (9.1%), tension pneumothorax (6%), commotio cordis (3%) [61] (S4 Appendix).

Prehospital thoracotomy was performed in 63 out of 915 patients with OHCA following blunt and penetrating trauma. However, only two patients survived to hospital discharge (5.6% vs 6.9%;p = 1) [61] (Table 4).

## Discussion

The main finding of this scoping review was inconsistency in reported survival outcome estimates of OHCA with confirmed etiologies in comparison with presumed cardiac (Utstein 2004) or medical (Utstein 2015) etiological classifications. This scoping review explored the reported survival outcomes following each confirmed etiology in comparison with presumed cardiac (Utstein 2004) or medical (Utstein 2015) classifications. This finding differs from the result of a prior systematic review that reported presumed cardiac etiology had higher 30-days survival outcome when compared to generic classification of non-traumatic etiologies (10.5% vs 6.7%; p < 0.001) [3]. A review of 27 population-based datasets in 2017 suggested that presumed cardiac as an etiological classification varied from 50–90% across datasets and suggested that this variability may affect outcome reporting [9,10].

When compared with the generic Utstein classifications (2004 and or 2015), studies reported better outcomes following OHCA for confirmed drug overdose and drowning. However, the likelihood of survival to discharge or 30-day survival following confirmed trauma, hanging, ICH, stroke, and respiratory diseases (broadly defined) was up to five times lower (Table 3). Thus, including a mix of etiologies within a cohort of patients may affect the reported outcomes in either direction.

A previous scoping review showed a discrepancy between initial etiological classification documented by EMS using Utstein 2004 or 2015 template and final etiologies recorded on medical or autopsy reports [8]. Defining inclusion criteria based on presumed cardiac (Utstein 2004), non-traumatic, or medical (Utstein 2015) etiologies may be too broad [3,7,65]. Any of these Utstein categories includes etiologically inconsistent patient cohorts who may or may not benefit from a specific treatment (e.g., trial intervention). Hence, etiology of OHCA should be considered as an essential inclusion criterion to recruit etiologically consistent patient cohorts or alternatively be considered as a prespecified intention to treat (ITT) analysis which may refine reported outcomes.

This scoping review has identified that a limited number of RCTs and observational studies that have been conducted to evaluate the effectiveness of prehospital cause-targeted interventions to treat reversible etiologies of OHCA (e.g., thrombolytic for some cardiac etiologies and PE, naloxone for drug overdose, alternative CPR strategies for drowning and respiratory illness, and prehospital interventions to treat underlying etiology of traumatic arrest) (Table 4). The few selected studies reported neutral outcomes, and the cohort was etiologically heterogeneous in most cases except for trauma. These findings may suggest why previous well-designed double blinded RCTs have not shown a significant difference [14,66–68]. These studies do suggest that prehospital cause-targeted interventions are feasible for reversible etiologies of OHCA in the prehospital setting. [55–57,60,61,66]. We may just have to refine our clinical tools to define etiology at the bedside prior to randomization.

Limited evidence and neutral outcomes following interventional trials might be attributed to difficulty in selecting the more likely etiology of OHCA that will respond to the intervention, small sample size, or difficulty in selecting patient cohorts with a similar etiology of cardiac arrest. Further research on circumstance-related factors of cardiac arrest might potentially help prehospital personnel establish more likely etiology of cardiac arrest. Providing a standardized case definition across data registries would enable researchers or data users to select etiologically consistent patient cohorts across all registries. Consistent patient cohorts by etiology may make the reported results more comparable across different registries.

This scoping review also demonstrated that a few studies with well-defined inclusion criteria based on etiology showed better survival outcome following thrombolytic treatment compared with studies that selected patients with presumed cardiac (Utstein 2004) etiologies or based on presenting rhythm [48,50,52,53]. However, the certainty of their results was low because there was no control or placebo group to compare the result with or small sample size. This finding suggests that etiologically well-defined inclusion criteria may result in better outcome data than out-of-hospital RCTs or observational studies evaluating prehospital cause-targeted intervention that selected etiologically heterogeneous patient cohorts [13,69,70].

Our review identified two studies that examined different bystander resuscitations (conventional CPR vs no CPR or compression only) for OHCA due to respiratory disease and drowning [44,59]. These studies suggested that conventional resuscitation resulted in better outcomes for patients with OHCA due to respiratory disease or drowning when compared to presumed cardiac (Utstein 2004) [44,59]. Our findings might be consistent with a subgroup analysis from a RCT which randomized OHCA patients regardless of the underlying etiology of cardiac arrest and reported better survival to hospital discharge following chest compression only versus conventional CPR (12.5% vs 11%; p = 0.31) [71]. After adjusting the denominator to include only cardiac etiology, chest compression only was superior to conventional CPR (survival to hospital discharge: 15.5% vs 12%;p = 0.09) [71]. In contrast, for non-cardiac etiologies, conventional CPR resulted in slightly better survival to discharge than compression only (7.2% vs 5%;p = 0.29) [71]. This better outcome following etiological stratification emphasizes the importance of ascertaining the etiology of cardiac arrest and potentially tailoring the resuscitation based on the underlying etiology of cardiac arrest. More trials with well-defined inclusion criteria are needed to further understand the impact of different bystander resuscitation on outcome of OHCA by etiologies.

### Limitation

This scoping review has some limitations that should be considered when interpreting the results. This review excluded gray literature and studies published in languages other than English which may result in the limited numbers of articles. Just one reviewer screened studies due to infeasibility of having the second reviewer and limited time. All the included studies were predominantly published in developed countries with data from resuscitation registries. No study has been found from developing countries which might be the result of restricting the search to English language. Our search also excluded pediatric patients with OHCA on the basis that the etiology, resuscitation practice, post arrest care, and outcome of OHCA in pediatric cohorts were quite different.

## Conclusion

This review highlights that the outcome of OHCA reported by confirmed etiologies is different across studies compared with outcome reported by presumed cardiac (Utstein 2004) or medical (Utstein 2015) etiological classifications. Outcomes of OHCA correctly attributed to drowning and drug overdose were higher than those reported with a generic etiological classification whereas the outcome following confirmed trauma, hanging, respiratory disease, and neurological etiologies may be up to five times lower than outcomes associated with presumed cardiac (Utstein 2004) or medical (Utstein 2015) etiological classifications. Prehospital interventional trials have predominantly resulted in neutral outcomes which may be due in part to difficulty in establishing the etiology of cardiac arrest and selecting etiologically homogeneous patient cohorts that most likely benefit from the proposed intervention. Further research might be needed to understand the role of contributing factors, triggers, or prodromal symptoms of OHCA, which may help prehospital emergency personnel to establish the more likely etiology of OHCA and tailor the interventions more appropriately. Consistency in etiological classification may lead to more comparable reporting across registries and may have an impact on randomized controlled trial outcomes evaluating cause-targeted interventions.

## Supporting information

S1 Appendix Preferred Reporting Items for Systematic reviews and Meta-Analyses extension for Scoping Reviews (PRISMA-ScR) Checklist. (DOCX)

S2 Appendix Search strategies and mesh terms used to conduct this scoping review. (DOCX)

S3 Appendix Summary of general characteristics of included articles evaluating survival outcome based on the etiology of out-of-hospital cardiac arrest (OHCA). (DOCX)

S4 Appendix Summary of general characteristics of included studies evaluating the cause-targeting interventions of out-of-hospital cardiac arrest (OHCA). (DOCX)
