# Supplementary material for: The outcome of out-of-hospital cardiac arrest based on the etiology of cardiac arrest; A scoping review
Source: PLoS One. 2025 Aug 11;20(8):e0330083. doi: 10.1371/journal.pone.0330083 (PMC12338839; doi:10.1371/journal.pone.0330083)
Supplement: S2 Appendix — (DOCX) [file pone.0330083.s002.docx]

**S2 Appendix: Search strategies and mesh terms used to conduct this scoping review**

| **All Ovid Medline <1946 – 28 June 2024>:**  1 exp *heart arrest/ep, et, sn 8584  2 OHCA.ti,ab. [Look for acronym in title and abstract] 3192  3 ("out-of-hospital" adj2 arrest).ti,ab,kf. 6920  4 ("out-of-hospital" adj2 cardiac).ti,ab,kf. 7219  5 ("out-of-hospital" adj2 heart).ti,ab,kf. 37  6 Death, Sudden, Cardiac/ep, et, sn 10428  7 (Sudden Cardiac adj2 Death).ti,ab,kf. 17577  8 or/1-7 33211  9 randomized controlled trial.pt. or randomized.mp. or randomised.mp. or placebo.mp. 1029121  10 8 not 9 30698  11 ambulance*.tw. 11491  12 emergency medical tech:.ti,ab,kf. 1250  13 EMT.ti,ab. 26287  14 first responder*.ti,ab,kf. 2702  15 paramedic.ti,ab,kf. 2880  16 exp *emergency medical services/ 99347  17 exp *emergency responders/ 9804  18 (out-of-hospital adj2 (treatment or diagnosis)).ti,ab,kf. 277  19 incident report*.ti,ab. 2324  20 witness.ti,ab. 6876  21 or/11-19 143228  22 (diagnosis or report).ti,ab,kf. or di.fs. 5226068  23 21 and 22 28129  24 (aetiology or etiology).ti,ab,kf. 301274  25 autops*.ti,ab,kf. 78187  26 ep.fs. [epidemiology as a subheading] 1878435  27 et.fs. [etiology. as a subheading] 2635211  28 exp causality/ 912940  29 cause of death/ 51948  30 (cause or causation).ti,ab,kf. 1083412  31 differential diagnosis/ 461611  32 epistry.af. 58  33 precipitated.contributing ti,ab,kf. 25626  34 presumed cause.mp. 257  35 (relative risk: or risks).tw. or cohort stud:.mp. 765592  36 or/23-35 6195716  37 exp mortality/ or mo.fs. 834257  38 exp Prognosis/ 1779927  39 prognosis.sh. or diagnosed.tw. or cohort:.mp. or predictor:.tw. or death.tw. or exp models, statistical/ 3029968  40 exp survival analysis/ 320015  41 survival.ti,ab,kf. 1045131  42 exp Treatment Outcome/ 1153843  43 37 or 38 or 39 or 40 or 41 or 42 4782420  44 10 and 36 and 43 18341  45 exp animals/ not humans.sh. 4909955  46 limit 44 to (adaptive clinical trial or address or autobiography or bibliography or biography or case reports or comment or dictionary or directory or duplicate publication or editorial or equivalence trial or evaluation study or "expression of concern" or festschrift or interactive tutorial or interview or lecture or legal case or legislation or letter or news or newspaper article or observational study, veterinary or patient education handout or periodical index or personal narrative or portrait or randomized controlled trial or randomized controlled trial, veterinary or retracted publication or "retraction of publication" or technical report or twin study or video-audio media or webcast) 3803  47 45 or 46 4913734  48 44 not 47 14139  **EBM Reviews - Cochrane Database of Systematic Reviews <2005 to June 28, 2024>**  Search Strategy:  --------------------------------------------------------------------------------  1 Out of hospital cardiac arrest.mp. [mp=title, short title, abstract, full text, keywords, caption text]  2 OHCA.ti.  3 (out-of-hospital adj3 arrest).mp.  4 (out-of-hospital adj3 cardiac).ti.  5 (out-of-hospital adj3 heart).ti.  6 Sudden cardiac death.ti.  7 1 or 2 or 3 or 4 or 5 or 6  **Embase Classic+Embase <1947 to 28 June 2024>**  Search Strategy:  --------------------------------------------------------------------------------  1 exp "out of hospital cardiac arrest"/  2 OHCA.ti,ab.  3 (out-of-hospital adj3 arrest).ti,ab,kw.  4 (out-of-hospital adj3 attack).ti,ab,kw.  5 (out-of-hospital adj3 heart).ti,ab,kw.  6 (out-of-hospital adj3 infarction).ti,ab,kw.  7 exp sudden cardiac death/  8 heart arrest/  9 cardiopulmonary resuscitation/ (121036)  10 (heart arrest or cardiopulmonary resuscitation or CPR).ti,ab,kw.  11 1 or 2 or 3 or 4 or 5 or 6 or 7 or 8 or 9 or 10  12 exp ambulance/  13 ambulance.ti,ab,kw.  14 (EMT or emergency medical tech* or paramedic or first respond*).ti,ab,kw.  15 exp *emergency medical services/  16 exp *emergency responders/  17 12 or 13 or 14 or 15 or 16  18 11 and 17  19 (aetiology or etiology).ti,ab,kw.  20 exp etiology/  21 exp causality/  22 (cause or causation).ti,ab,kw.  23 exp coroner/ (3041)  24 (coroner* or coronial or medical examiner* or death certificate*).ti,ab,kw.  25 data collection.mp.  26 ep.fs.  27 et.fs.  28 exp epidemiology/  29 epidemiology.ti,ab,kw.  30 epistry.af.  31 factual database*.ti,ab,kw.  32 information processing/  33 precipitated.tw.  34 presumed cause.mp.  35 prognosis.sh. or diagnosed.tw. or cohort:.mp. or predictor:.tw. or death.tw. or exp models, statistical/ [HIRU prognosis]  36 registries/ or registr*.ti,ab,kw.  37 exp retrospective study/  38 survival analysis/  39 survival.tw.  40 or/19-39  41 18 and 40  42 limit 41 to (human and english language)  43 remove duplicates from 42 |
| --- |
