# Supplementary material for: The outcome of out-of-hospital cardiac arrest based on the etiology of cardiac arrest; A scoping review
Source: PLoS One. 2025 Aug 11;20(8):e0330083. doi: 10.1371/journal.pone.0330083 (PMC12338839; doi:10.1371/journal.pone.0330083)
Supplement: S3 Appendix — (DOCX) [file pone.0330083.s003.docx]

**S3 Appendix: Summary of general characteristics of included articles evaluating survival outcome based on the etiology of out-of-hospital cardiac arrest (OHCA)**

| **Author** | **Year** | **Study design** | **Country** | **Source of initial etiology** | **Source of final etiology** | **Reported outcome** |
| --- | --- | --- | --- | --- | --- | --- |
| **Drug overdose** | | | | | | |
| Koller, et al ^[^[^1^](#_ENREF_1)^]^ | 2014 | Observational | USA | ROC (Utstein style) ^ | Medical record | ROSC, Hospital admission and Discharge |
| Elmer, et al ^[^[^2^](#_ENREF_2)^]^ | 2015 | Observational | USA | Emergency record ^ | Medical record | ROSC, Hospital discharge, Favorable neurological outcome |
| Salcido, et al ^[^[^3^](#_ENREF_3)^]^ | 2016 | Observational | USA | ROC (Utstein style) ^ | Medical record | ROSC, Hospital admission, Discharge |
| Orkins, et al ^[^[^4^](#_ENREF_4)^]^ | 2017 | Observational | Canada | Epistry (Utstein style) ^ | Medical record | ROSC, Hospital admission and discharge, Favorable neurological outcome |
| Yogeswaran, et al ^[^[^5^](#_ENREF_5)^]^ | 2023 | Observational | USA | EMS (Utstein style) ^ | Medical and autopsy report | Hospital discharge, Favorable neurological outcome |
| Shekhar, et al ^[^[^6^](#_ENREF_6)^]^ | 2024 | Observational | USA | CARES (Utstein style) ^ | Not reported | Favorable neurological outcome |
| **Drowning** | | | | | | |
| Claesson, et al ^[^[^7^](#_ENREF_7)^]^ | 2008 | Observational | Sweden | Swedish cardiac arrest registry (Utstein style) | Other sources not used | Hospital admission, 30-day survival |
| Grmec, et al ^[^[^8^](#_ENREF_8)^]^ | 2009 | Observational | Slovenia | EMS data (Utstein style) | Medical record | ROSC, Hospital admission and discharge, Favorable neurological outcome |
| Buick, et al ^[^[^9^](#_ENREF_9)^]^ | 2011 | Observational | Canada | Epistry (Utstein style) | Medical record | Hospital admission and discharge |
| **Trauma** | | | | | | |
| Deasy, et al ^[^[^10^](#_ENREF_10)^]^ | 2011 | Observational | Australia | VACAR (Utstein style) * | Medical record | ROSC, Hospital discharge |
| Beck,B, et al ^[^[^11^](#_ENREF_11)^]^ | 2016 | Observational | Australia | SJA-WA (Utstein style) * | Medical record | Hospital admission and discharge |
| Barnard, et al ^[^[^12^](#_ENREF_12)^]^ | 2018 | Observational | UK | EEAST (Utstein style) ** | Not reported | Hospital admission and discharge |
| Djarv, et al ^[^[^13^](#_ENREF_13)^]^ | 2018 | Observational | Sweden | SRCR (Utstein style) * | Not reported | 30-day survival |
| Escutnaire, et al ^[^[^14^](#_ENREF_14)^]^ | 2018 | Observational | France | EMS data (Utstein style) * | Not reported | 30-day survival |
| Wolthers, et al ^[^[^15^](#_ENREF_15)^]^ | 2023 | Observational | Denmark | Danish cardiac arrest registry (Utstein style) * | Adjudicated by three independent reviewers | ROSC, hospital admission, 30-day survival |
| **Hanging** | | | | | | |
| Deasy, et al ^[^[^16^](#_ENREF_16)^]^ | 2013 | Observational | Australia | VACAR (Utstein style) | Medical record | ROSC, Hospital admission and discharge |
| Shin, et al ^[^[^17^](#_ENREF_17)^]^ | 2014 | Observational | South Korea | KORHN (Utstein style) | Medical record | Hospital discharge, Favorable neurological outcome |
| **Respiratory Disease** | | | | | | |
| Herlitz, et al ^[^[^18^](#_ENREF_18)^]^ | 1996 | Observational | Sweden | EMS data*** | Medical and autopsy report | Hospital discharge |
| Fukuda, et al ^[^[^19^](#_ENREF_19)^]^ | 2015 | Observational | Japan | Utstein registry | Not reported | ROSC, 30-day survival, Favorable neurological outcome |
| Orban, et al ^[^[^20^](#_ENREF_20)^]^ | 2018 | Observational | France | EMS data (Utstein style) **** | ICU record | Favorable neurological outcome |
| **Intracranial hemorrhage and stroke** | | | | | | |
| Shin, et al ^[^[^21^](#_ENREF_21)^]^ | 2016 | Observational | South Korea | OHCA registry (Utstein style) | Medical record | ROSC, hospital admission and discharge, Favorable neurological outcome |
| Fukuda, et al ^[^[^22^](#_ENREF_22)^]^ | 2016 | Observational | Japan | Utstein registry | Medical and coroner reports | ROSC,30-day survival, Favorable neurological outcome |

**CARES:** Cardiac Arrest Registry to Enhance Survival**. CPR**: Cardiopulmonary resuscitation. **EEAST:** East of England Ambulance Service**. EMS:** Emergency medical service**. ICH**: Intracranial hemorrhage. **ICU**: Intensive care unit. **KORHN**: Korean hypothermia network. **ROSC**: Return of spontaneous circulation. **OHCA**: Out-of-hospital cardiac arrest. **ROC**: Resuscitation outcomes consortium. **SJA-WA**: St John Ambulance Western Australia **SRCR:** Swedish registry of cardiopulmonary resuscitation **VACAR**: Victorian ambulance cardiac arrest registry

^OD-OHCA was presumed based on EMS notes, prior history of drug use, patient characteristics (e.g. young age, asystolic rhythm), witnessed overdose or drug paraphernalia and confirmed based on toxicology test

*Traumatic OHCA was defined as blunt, penetrating, burning injury, traffic accident (pedestrian or driver), or falling from heights according to Utstein definition.

**Non-traumatic cardiac arrest defined as all medical, asphyxiation, drowning, and electrocution. Traumatic cardiac arrest defined as any cardiac arrest resulted from external kinetic energy.

*** The diagnosis of obstructive pulmonary disease was presumed based on clinical history, signs of obstruction at ventilation, and autopsy findings

**** The following diagnoses were considered as a respiratory etiology of OHCA: asthma attack, pneumonia, aspiration, pulmonary embolism, chronic obstructive pulmonary disease (COPD), hanging, and drowning

**References:**

1. Koller AC, Salcido DD, Callaway CW, Menegazzi JJ. Resuscitation characteristics and outcomes in suspected drug overdose-related out-of-hospital cardiac arrest. Resuscitation. 2014;85(10):1375-9.

2. Elmer J, Lynch MJ, Kristan J, Morgan P, Gerstel SJ, Callaway CW, et al. Recreational drug overdose-related cardiac arrests: break on through to the other side. Resuscitation. 2015;89:177-81.

3. Salcido DD, Torres C, Koller AC, Orkin AM, Schmicker RH, Morrison LJ, et al. Regional incidence and outcome of out-of-hospital cardiac arrest associated with overdose. Resuscitation. 2016;99:13-9.

4. Orkin AM, Zhan C, Buick JE, Drennan IR, Klaiman M, Leece P, et al. Out-of-hospital cardiac arrest survival in drug-related versus cardiac causes in Ontario: A retrospective cohort study. PloS one. 2017;12(4):e0176441.

5. Yogeswaran V, Drucker C, Kume K, Poel A, Yarid N, Leyde S, et al. Presentation and Outcomes of Adults With Overdose-Related Out-of-Hospital Cardiac Arrest. JAMA Network Open. 2023;6(11):e2341921.

6. Shekhar AC, Nathanson BH, Mader TJ, Coute RA. Cardiac Arrest Following Drug Overdose in the United States: An Analysis of the Cardiac Arrest Registry to Enhance Survival. J Am Heart Assoc. 2024;13(3):e031245.

7. Claesson A, Svensson L, Silfverstolpe J, Herlitz J. Characteristics and outcome among patients suffering out-of-hospital cardiac arrest due to drowning. Resuscitation. 2008;76(3):381-7.

8. Grmec S, Strnad M, Podgorsek D. Comparison of the characteristics and outcome among patients suffering from out-of-hospital primary cardiac arrest and drowning victims in cardiac arrest. International Journal of Emergency Medicine. 2009;2(1):7-12.

9. Buick JE, Lin S, Rac VE, Brooks SC, Kierzek G, Morrison LJ. Drowning: an overlooked cause of out-of-hospital cardiac arrest in Canada. CJEM. 2014;16(4):314-21.

10. Deasy C, Bray J, Smith K, Harriss L, Morrison C, Bernard S, et al. Traumatic out-of-hospital cardiac arrests in Melbourne, Australia. Resuscitation. 2011.

11. Beck B, Tohira H, Bray JE, Straney L, Brown E, Inoue M, et al. Trends in traumatic out-of-hospital cardiac arrest in Perth, Western Australia from 1997 to 2014. Resuscitation. 2016;98:79-84.

12. Barnard EBG, Sandbach DD, Nicholls TL, Wilson AW, Ercole A. Prehospital determinants of successful resuscitation after traumatic and non-traumatic out-of-hospital cardiac arrest. Emergency medicine journal : EMJ. 2019;36(6):333-9.

13. Djarv T, Axelsson C, Herlitz J, Stromsoe A, Israelsson J, Claesson A. Traumatic cardiac arrest in Sweden 1990-2016 - a population-based national cohort study. Scandinavian journal of trauma, resuscitation and emergency medicine. 2018;26(1):30.

14. Escutnaire J, Genin M, Babykina E, Dumont C, Javaudin F, Baert V, et al. Traumatic cardiac arrest is associated with lower survival rate vs. medical cardiac arrest – Results from the French national registry. Resuscitation. 2018;131:48-54.

15. Wolthers SA, Jensen TW, Breindahl N, Milling L, Blomberg SN, Andersen LB, et al. Traumatic cardiac arrest – a nationwide Danish study. BMC Emergency Medicine. 2023;23(1).

16. Deasy C, Bray J, Smith K, Bernard S, Cameron P, Committee VS. Hanging-associated out-of-hospital cardiac arrests in Melbourne, Australia. Emergency medicine journal : EMJ. 2013;30(1):38-42.

17. Shin J, Lee H, Kim J, Kim J, Choi S, Jeung K, et al. Outcomes of hanging-induced cardiac arrest patients who underwent therapeutic hypothermia: a multicenter retrospective cohort study. Resuscitation. 2014;85(8):1047-51.

18. Herlitz J, Rosenfelt M, Bang A, Axelsson A, Ekstrom L, Wennerblom B, et al. Prognosis among patients with out-of-hospital cardiac arrest judged as being caused by deterioration of obstructive pulmonary disease. Resuscitation. 1996;32(3):177-84.

19. Fukuda T, Fukuda-Ohashi N, Doi K, Matsubara T, Yahagi N. Effective pre-hospital care for out-of-hospital cardiac arrest caused by respiratory disease. Heart, lung & circulation. 2015;24(3):241-9.

20. Orban J-C, Truc M, Kerever S, Novain M, Cattet F, Plattier R, et al. Comparison of presumed cardiac and respiratory causes of out-of-hospital cardiac arrest. Resuscitation. 2018;129:24-8.

21. Shin J, Kim K, Lim YS, Lee HJ, Lee SJ, Jung E, et al. Incidence and clinical features of intracranial hemorrhage causing out-of-hospital cardiac arrest: a multicenter retrospective study. The American journal of emergency medicine. 2016;34(12):2326-30.

22. Fukuda T, Ohashi-Fukuda N, Kondo Y, Sera T, Doi K, Yahagi N. Epidemiology, Risk Factors, and Outcomes of Out-of-Hospital Cardiac Arrest Caused by Stroke: A Population-Based Study. Medicine. 2016;95(14):e3107.
