# Supplementary material for: The outcome of out-of-hospital cardiac arrest based on the etiology of cardiac arrest; A scoping review
Source: PLoS One. 2025 Aug 11;20(8):e0330083. doi: 10.1371/journal.pone.0330083 (PMC12338839; doi:10.1371/journal.pone.0330083)
Supplement: S4 Appendix — (DOCX) [file pone.0330083.s004.docx]

**S4 Appendix: Summary of general characteristics of included studies evaluating the cause-targeting interventions of out-of-hospital cardiac arrest (OHCA)**

| **Author** | **Year/**  **Country** | **Study design** | **Included presumed etiologies** | **Number of patients** | **Accuracy of initial diagnosis: N(%)** | **Source of initial etiology** | **Source of final etiology** | **Reported outcome** |
| --- | --- | --- | --- | --- | --- | --- | --- | --- |
| **Thrombolysis therapy** | | | | | | | | |
| **1-Presumed cardiac etiologies** | | | | | | | | |
| Abu-Laban, et al ^[^[^1^](#_ENREF_1)^]^ | 2002  Canada | Randomized controlled trial | OHCA with PEA | 233 | CVD:25^*^  MI:9  AD:4  PE:1 | EMS data  (Utstein style) | Autopsy report | ROSC, Hospital admission and discharge |
| Bottiger,et al ^[^[^2^](#_ENREF_2)^]^ | 2008  Multicentre | Double-blind randomized controlled trial | Presumed cardiac | 1050 | Not reported | EMS data  (Utstein style) | Not reported | ROSC, Hospital admission and discharge, Favorable neurological outcome |
| Voipio, et al ^[^[^3^](#_ENREF_3)^]^ | 2001  Finland | Observational | AMI | 68 | 64 | EMS data  (Utstein style) | MICU, Autopsy report | ROSC, Favorable neurological outcome |
| Renard, et al ^[^[^4^](#_ENREF_4)^]^ | 2001  France | Observational | Non-traumatic | 1261 | 13^$^ | EMS data  (Utstein style) | Autopsy report | Hospital admission |
| Bottiger, et al ^[^[^5^](#_ENREF_5)^]^ | 2001  Germany | Observational | Unsuccessful CPR | 90 | Not reported | EMS data  (Utstein style) | Not reported | ROSC, Hospital admission and discharge |
| Lederer, et al ^[^[^6^](#_ENREF_6)^]^ | 2001  Austria | Observational | Non-traumatic | 401 | Not reported | EMS data  (Utstein style) | Medical record | ROSC, Hospital discharge |
| Arntz, et al ^[^[^7^](#_ENREF_7)^]^ | 2008  Germany | Observational | AMI (STEMI) | 53 | 30  (56%) | EMS data  (Utstein style) | Autopsy and medical record | Hospital admission and discharge |
| **2- Pulmonary Embolism (PE)** | | | | | | | | |
| Kurkciyan, et al ^[^[^8^](#_ENREF_8)^]^ | 2000  Austria | Observational | Pulmonary embolism^@^ | 20 | 20  (100%) | Emergency medicine  (Utstein style) | Autopsy report  Medical chart^@^ | ROSC, Hospital discharge |
| Bougouin, et al ^[^[^9^](#_ENREF_9)^]^ | 2017  France | Observational | Pulmonary embolism | 82 | 74  (90%) | SDEC ^@@^  (Utstein style) | 2 investigators’ adjudication ^@^ | Hospital discharge |
| Javaudin, et al ^[^[^10^](#_ENREF_10)^]^ | 2019  France | Observational | Pulmonary embolism | 246 | Not reported | OHCA registry (Utstein style) | Medical record^@^ | 30-day survival,  Favorable neurological outcome |
| **Naloxone** | | | | | | | | |
| Saybolt, et al ^[^[^11^](#_ENREF_11)^]^ | 2010  USA | Observational | Presumed opioid overdose | 36 | Not reported | ALS response data | Not reported | Hospital admission and discharge |
| **Different resuscitation strategies** | | | | | | | | |
| Fukuda,et al ^[^[^12^](#_ENREF_12)^]^ | 2015  Japan | Observational | Respiratory diseases** | 7,071 | Not reported | All Japan Utstein registry | Not reported | ROSC.30-day survival, Favorable neurological outcome |
| Fukuda, et al ^[^[^13^](#_ENREF_13)^]^ | 2019  Japan | Observational | Drowning** | 5,121 | Not reported | All Japan Utstein registry | Medical chart | ROSC,30-day survival, Favorable neurological outcome |
| **Prehospital interventions for traumatic OHCA** | | | | | | | | |
| Houwen, et al ^[^[^14^](#_ENREF_14)^]^ | 2021  Netherland | Observational | Trauma*** | 915 | 915**** | HEMS medical data (Utstein style) | Hospital chart | Hospital discharge |
| Ohlen, et al ^[^[^15^](#_ENREF_15)^]^ | 2022  Sweden | Observational | Trauma*** | 256 | 256 | Swedish OHCA and trauma registry  (Utstein style) | Not reported | 30-day survival |
| Smida, et al ^[^[^16^](#_ENREF_16)^]^ | 2023  USA | Observational | Trauma*** | 4942 | Not reported | EMS database  (Utstein style) | Not reported | ROSC |
| Nagasawa, et al ^[^[^17^](#_ENREF_17)^]^ | 2023  Japan | Observational | Trauma*** | 722 | Not reported | JSAS-R  (Utstein 2015) | Not reported | ROSC |
| Wolthers, et al ^[^[^18^](#_ENREF_18)^]^ | 2023  Denmark | Observational | Trauma*** | 223 | Not reported | EMS and HEMS databases (Utstein2015) | Not reported | ROSC, 30-day survival |

**AD:** Aortic dissection**. ALS:** Advanced life support. **AMI**: Acute myocardial infarction. **CPR**: Cardiopulmonary resuscitation. **CVD:** Cardiovascular disease**. EMS:** Emergency medical service. **HEMS:** Helicopter Emergency Medical Services. **JSAS-R:** Japanese Society for Aeromedical Services.  **MI**: Myocardial infarction. **MICU:** Mobile intensive care unit. **OHCA**: Out-of-hospital cardiac arrest**. PE**: Pulmonary embolism. **PEA**: Pulseless electrical activity. **RCT**: Randomized controlled trial. **ROSC:** Return of spontaneous circulation. **SDEC:** Sudden death expertise centre. **STEMI**: ST elevation myocardial infarction.

*Reported based on the number of patients who underwent autopsy. Autopsy was performed for 42 patients.

^$^Autopsy was performed for 13 patients and the diagnosis was confirmed in all autopsied cases.

^@^ A tentative diagnosis of PE was based on medical history, symptoms, ECG changes (including, right bundle branch block, inverted T in V1-5, S1Q3, and complete heart block) prior to cardiac arrest or after ROSC. The diagnose was determined through transthoracic or transesophageal echo cardiogram, CT scan, ventilation-perfusion scan, or autopsy.

^@@^ The etiology of PE was presumed based on Wells rule =>2 and simplified Geneva score>= 3.

** The etiology of OHCA was established by emergency physician collaborating with EMS based on witness information, clinical presentation, medical history, and physical examination.

*** Trauma was defined as blunt, penetrating, burning injury, traffic accident (pedestrian or driver), or falling from heights according to Utstein definition.

****Suspected causes of out-of-hospital traumatic cardiac arrest were reported for 36 survivors, including hypoxia due to brain injury or obstructive airway (45%), Hypovolemia (24%), cardiac tamponade (12%), hypoxia due to other causes (9%), tension pneumothorax (6%), and commotio cordis (3%).

**References**

1. Abu-Laban RB, Christenson JM, Innes GD, Van Beek CA, Wanger KP, McKnight RD, et al. Tissue Plasminogen Activator in Cardiac Arrest with Pulseless Electrical Activity. New England Journal of Medicine. 2002;346(20):1522-8.

2. Böttiger BW, Arntz H-R, Chamberlain DA, Bluhmki E, Belmans A, Danays T, et al. Thrombolysis during Resuscitation for Out-of-Hospital Cardiac Arrest. New England Journal of Medicine. 2008;359(25):2651-62.

3. Voipio V, Kuisma M, Alaspaa A, Manttari M, Rosenberg P. Thrombolytic treatment of acute myocardial infarction after out-of-hospital cardiac arrest. Resuscitation. 2001;49(3):251-8.

4. Renard A, Verret C, Jost D, Meynard J-B, Tricehreau J, Hersan O, et al. Impact of fibrinolysis on immediate prognosis of patients with out-of-hospital cardiac arrest. Journal of thrombosis and thrombolysis. 2011;32(4):405-9.

5. Böttiger BW, Bode C, Kern S, Gries A, Gust R, Glätzer R, et al. Efficacy and safety of thrombolytic therapy after initially unsuccessful cardiopulmonary resuscitation: a prospective clinical trial. The Lancet. 2001;357(9268):1583-5.

6. Lederer W, Lichtenberger C, Pechlaner C, Kroesen G, Baubin M. Recombinant tissue plasminogen activator during cardiopulmonary resuscitation in 108 patients with out-of-hospital cardiac arrest. Resuscitation. 2001;50(1):71-6.

7. Arntz HR, Wenzel V, Dissmann R, Marschalk A, Breckwoldt J, Muller D. Out-of-hospital thrombolysis during cardiopulmonary resuscitation in patients with high likelihood of ST-elevation myocardial infarction. Resuscitation. 2008;76(2):180-4.

8. Kurkciyan I, Meron G, Sterz F, Janata K, Domanovits H, Holzer M, et al. Pulmonary embolism as a cause of cardiac arrest: presentation and outcome. Archives of internal medicine. 2000;160(10):1529-35.

9. Bougouin W, Marijon E, Planquette B, Karam N, Dumas F, Celermajer DS, et al. Pulmonary embolism related sudden cardiac arrest admitted alive at hospital: Management and outcomes. Resuscitation. 2017;115:135-40.

10. Javaudin F, Lascarrou J-B, Le Bastard Q, Bourry Q, Latour C, De Carvalho H, et al. Thrombolysis During Resuscitation for Out-of-Hospital Cardiac Arrest Caused by Pulmonary Embolism Increases 30-Day Survival: Findings From the French National Cardiac Arrest Registry. Chest. 2019;156(6):1167-75.

11. Saybolt MD, Alter SM, Dos Santos F, Calello DP, Rynn KO, Nelson DA, et al. Naloxone in cardiac arrest with suspected opioid overdoses. Resuscitation. 2010;81(1):42-6.

12. Fukuda T, Fukuda-Ohashi N, Doi K, Matsubara T, Yahagi N. Effective pre-hospital care for out-of-hospital cardiac arrest caused by respiratory disease. Heart, lung & circulation. 2015;24(3):241-9.

13. Fukuda T, Ohashi-Fukuda N, Hayashida K, Kondo Y, Kukita I. Bystander-initiated conventional vs compression-only cardiopulmonary resuscitation and outcomes after out-of-hospital cardiac arrest due to drowning. Resuscitation. 2019;145:166-74.

14. Houwen T, Popal Z, de Bruijn MAN, Leemeyer AMR, Peters JH, Terra M, et al. Outcomes after Prehospital Traumatic Cardiac Arrest in the Netherlands: a Retrospective Cohort Study. Injury. 2021.

15. Ohlen D, Hedberg M, Martinsson P, von Oelreich E, Djarv T, Jonsson Fagerlund M. Characteristics and outcome of traumatic cardiac arrest at a level 1 trauma centre over 10 years in Sweden. Scandinavian journal of trauma, resuscitation and emergency medicine. 2022;30(1):54.

16. Smida T, Price BS, Scheidler J, Crowe R, Wilson A, Bardes J. Stay and play or load and go? The association of on-scene advanced life support interventions with return of spontaneous circulation following traumatic cardiac arrest. European Journal of Trauma and Emergency Surgery. 2023;49(5):2165-72.

17. Nagasawa H, Omori K, Muramatsu K-I, Takeuchi I, Ohsaka H, Ishikawa K, et al. Outcomes of prehospital traumatic cardiac arrest managed by helicopter emergency medical service personnel in Japan: a registry data analysis. International Journal of Emergency Medicine. 2023;16(1).

18. Wolthers SA, Breindahl N, Jensen TW, Holgersen MG, Møller TP, Blomberg SNF, et al. Prehospital interventions and outcomes in traumatic cardiac arrest: a population-based cohort study using the Danish Helicopter Emergency Medical Services data. European Journal of Emergency Medicine. 9900.
